# The Nature and Treatment of Gout

**Published:** 1846-01

**Authors:** 


THE NATURE AND TREATMENT OF GOUT. By *William Henry Robertson,* M.D. Physician to the Buxton Bath Charity. 8vo. pp. 372. London, 1845. Churchill.

THOSE, who are accustomed to the task of reviewing, know full well how much more easy it is to analyse and report upon certain works than upon others. In many cases where some difficulty is experienced, this arises not so much from the abstruse and unattractive nature of the subject discussed, nor yet from the extent and intricacy of the details that are necessarily advanced, as from the want of a lucid exposition and an orderly arrangement on the part of the author. Some men have the happy tact of enucleating every subject that they take in hand; they quickly perceive where the germ or kernel is lodged; and, without spending much time in minutely examining each separate envelope with which it may be invested, they proceed to the core at once; and this they do so cleverly that others are surprised at their own want of skill in waiting to be taught. But the thing is not so easy as might be imagined; for the dexterity, that is so much admired, is the result not of inconsiderate rashness, but of previous patient and elaborate practice. He, who can get quickly at the pith and substance of a work that he reads, has been at one time, we may rest assured, a laborious and pains-taking student: his very quickness is the offspring of past toil. This prompt perception of the truth is a most important qualification in any one who aims at being a lucid and instructive writer; and it is from the too common want of it that so many of our modern authors fail in attaining the end to which they aspire: viz. that of impressing their own thoughts and sentiments upon the minds of other people.

There are few terms in medical language, that have given rise to greater errors, in practice as well as in theory, than that of Gout. What definition shall we give of it?—we hesitate to reply; for there is none that will bear the test of criticism. Take, for example, that recently proposed by Dr. Copland, one of the most practised writers of the day. *Constitutional disorder, giving rise to a specific form of inflammation; often favoured by original or hereditary constitution; appearing after puberty, chiefly in the male sex; returning after intervals; generally preceded by, or alternating with, disorder of the digestive or other internal organs; and characterised by affection of the first joint of the great toe, by nocturnal exacerbations and morning remissions, and by vascular plethora; various joints or parts becoming affected after repeated attacks, without passing into suppuration.*

Now when we say that the disease is *characterised by an affection of the first joint of the great toe*, the remark is perfectly true as regards Podagra, or, as it is frequently called, Regular Gout; but certainly not as regards various morbid states to which the term gout is universally applied. The misfortune is, that the same name is used to designate very different maladies; the confusion having arisen in consequence of a *generic* being substituted for a *specific* appellation. When we talk of gout in the stomach we mean a very different—different not only in its locality but also, at least often, in its very nature—disease from gout as it manifests itself in the feet or hands. The one may be, and very frequently is, a purely spasmodic malady; while the other is invariably attended with a greater or less amount of inflammatory action. To call, therefore, Gout an inflammation—even although we indicate its peculiarity by adding the epithet *specific* to it—is surely not correct; seeing that various morbid states, recognised as different forms or manifestations of gout, are unquestionably not of a phlogistic character. In place of regarding Gout as an individual and substantive disease, it should be viewed as a diseased condition of the system; for, in truth, it is a cachexia, or dyscrasis, or morbid diathesis quite as much as either Scrofula or Scurvy. No one would think of defining Scrofula by describing any one of its numerous and diversified forms; nor yet would it be ranked among the Phlegmasiæ; and yet we need not say how very frequent, alas! scrofulous inflammation is known to be. But then, inflammation is not the only way in which Scrofula manifests its existence; and he indeed knows little of the practice of his profession who would apply the same remedies to this disease (inflammation), when it exists in a scrofulous, as when it occurs in a healthy individual.

Dr. Robertson has fully appreciated the constitutional nature of gout, and he labours to establish the position in various parts of his volume. We adduce the following passage, one out of many, in which he alludes to the influence of hereditary predisposition, for more reasons than one.

"Gout rarely becomes hereditary, without some portion of cachectic predisposition being transmitted at the same time. A man rarely acquires the gouty habit, without rendering his system more or less cachectic. The amount of cachexia will depend on numberless circumstances. Cachexia, of course, most frequently occurs unconnected with gout; but it may be doubted, whether gout ever occurs without more or less of cachexia. To the production of gout, circumstances are necessary that are not indispensable to the production of cachexia—whether simple cachexia, or this in combination with other morbid conditions. The breathing an impure air for a long time, whether vitiated by miasmata, or putrescence, or imperfect ventilation,—excessive venery, with or without the disease so frequently consequent on its indulgence,—abuse of fermented liquors,—excess or deficiency of nourishment, or the being confined to too few articles of diet,—are some of the principal causes of cachexia, to which various superadded circumstances give a peculiar form and character. Whatever has the effect of superinducing plethora,—of rendering the supply of alimentary matters in the blood greater than their expenditure,—may probably have the effect of inducing whatever is the proximate cause of gout; or this may be modified or counteracted by some other agent, favouring the production of some other disease in its stead. In confirmation of these views, it is not uncommon to find cachexia traceable in a family history in some one form, as in that of severe irregular gout, producing in the different individuals of the existing race different cachectic diseases. One may be phthisical, a second have glandular disease of strumous character, a third may have articular rheumatism of cachectic character, a fourth may have irregular gout, a fifth scrofulous disease of the joints, and a sixth have died in infancy of mesenteric or hydrocephalic disease; and all these cases may be apparently traceable to the original influence or taint. It might perhaps, then, not be saying too much to affirm, that gout, in all its forms and degrees, is connected more or less with cachexia; and it is at all events true, that there is always this connection in the worse forms of gout." P. 173.

There is much that is not only ambiguous, but of very questionable accuracy, in this extract. The Cachexia, of which Dr. R. speaks, seems to the reader as if it were a something that is superadded and accessory, rather than as essential, to "the gouty habit." According to our view of the question, this habit—in other words, the constitution of the body in which gouty maladies are apt to occur—necessarily implies an existing dyscrasis or unhealthy condition of the blood and its secretions. The cachexia, which is induced by breathing a vitiated atmosphere or by penury of nourishing food, has nothing to do with that which is characteristic of gout; and as to endeavouring to establish any family alliance between gouty and scrofulous disease, the observation of most medical men is surely against it.

There is one paragraph in the preceding extract to which we would especially invite the reader's attention; it is that where allusion is made to the immediate influence of Plethora in "inducing whatever is the proximate cause of gout." Now this, we may remark, is the key-note that pervades the whole performance of our author from beginning to end. It is variously modulated and expressed in different sections of the work, but still it may be recognised in every part, giving, as it were, a general tone and character to the entire composition. To prevent all mistake as to the meaning of the term, we shall select one or two passages where it is more particularly described.

"Using the word plethora to mean that condition in which the blood is probably excessive in point of quantity, and deficient in its proportion of serum, the red particles being in excess, and redundantly charged with fibrine, it is, as far as we know, the predisponent of gout; and it may not be too much to say, that every person affected with it, is more or less liable to gout; a liability which may be added to, or diminished, by many circumstances, and which may never end in the disease, but terminate in some other ailment, or may be relieved or diminished by change of habits, or by the alterations consequent on advancing life, till the plethoric state gradually passes away, with its risks and its dangers." P. 51.

In another passage, Dr. R. thus expresses himself:—

"From the account of the remote causes of the gouty diathesis it will have been gathered, that it is by inducing or adding to a plethoric habit, that they produce this diathesis, and that plethora is probably the predisposing cause of gout. It should be remembered, that plethora is not confined to those cases in which much is added to the fluids and solids of the system, but includes those cases in which less of the solids and fluids is expended than is received; and that consequently a man of spare habit, who is abstemious and temperate, may, by taking too little or too much sleep, or by sleeping at irregular periods and at variable times, or by long-continued sedentary habits, or by taking exercise at irregular periods and in varying amount, or by using his mind unduly, produce in his system the same result, as the bon-vivant and intemperate arrive at by an opposite route. This explains the apparent anomaly of people so different as the sedentary, spare, pale-faced student, and the bloated and unctuous high and full feeder, and the gross, and rubicund, and pimple-faced sot, should so often be victimised by the different diseases to which plethora lays the train; and it serves to give intelligibility, correctness, and simplicity to the various means advised for the prevention of gout." P. 39.

This doctrine of the plethoric origin of Gout—a doctrine, by the bye, that has been so elaborately worked out by Dr. Barlow, of Bath, although we find no allusion to his writings in the present work—leads our author occasionally into some very untenable positions. We have already found him asserting that, "it may not be too much to say that every person affected with it (plethora) is more or less liable to gout;"—an assertion which must surely have dropped from his pen by sheer inadvertence—and in another place, he attributes the increasing tendency to the recurrence of the disease, after every successive fit, to the circumstance that "Plethora is more easily kept up, and more easily reproduced than originally engendered." We do not at present dispute this fact; but we certainly cannot admit its application as our author intends; for, is it not an occurrence of very frequent observation, that the attacks of Gouty disease often become more and more frequent in their returns, and more severe in their character, just in proportion as the patient's flesh and strength decay?

True it is that Dr. Robertson, in other passages of his work, has very materially qualified the above proposition, and that, in one of these, he ascribes the increasing inveteracy of the malady rather to "a loss of tone or vital contractility in the vessels of the (affected) part, or to some mysterious and undiscovered cause," than to an actually plethoric state of the body; yet he is continually making reference to an overcharged and redundant quantity of nutrimentary matters in the blood; and, on one occasion, he goes so far as to compare the influence of the gouty habit to that "of periodical losses of blood, whether naturally or artificially produced, the consequence of which we know to be increased action of the formative, and diminished action of the destructive, organs, and an eventual addition to any tendency to fulness of the general system."

But let us not be unfair to our author by omitting to mention that it is chiefly in the light of a *predisposing* cause of the disease, that he attaches so much importance to vascular Plethora. Yet, even in this point of view, many will be of opinion that he has very much over-rated its influence—provided always we are to comprehend, under the generic term of Gout, the various morbid states that are generally recognised to belong to it. That an over-replete state of the vessels, with a rich and highly-animalised blood, will render a person more liable to *arthritic inflammations* than he would be if he were of a more spare habit of body, we may readily admit, without recognising the truth of the hypothesis which Dr. R. labours so perseveringly to establish.

But we shall not dwell more upon this topic at present, as it may again come under our consideration, when we allude to the diagnosis between Rheumatism and Gout. Meanwhile we wish to make a few remarks upon some of the other predisposing or remote causes of the latter disease; and of these, more particularly upon the alleged influence of hereditary liability, and of inordinate exercise of the intellectual powers. Let us first hear what our author says upon these matters. Almost at the very commencement of the Introductory Chapter of his work, we read that—

"It is chiefly the highest ranks of life, and the most thinking classes of the community, that are attacked by this disease; the liability to its influence does not extend to the masses of the people; but it is with few exceptions, confined to those who have raised themselves, or been raised by the exertions of their fathers, above the level of their fellows. The higher the rank, the more pure and noble the blood, the greater the powers of mind, and the more those powers are used, the nearer the man is to the time of life when his intellect is the most vigorous, the more liable is he to become the victim of this disease." P. 2.

Again, at page 195, after a high-wrought picture of the miserable condition of many a gouty old invalid, we are presented with the following somewhat grandiloquent passage.

"We find the man of high and noble feelings, of pure and mighty thinkings, the highly intellectual and moral, the little sensual, the highly-born, or the self-ennobled children of the world,—the deep and close thinker,—the man of letters and hard study,—the successful soldier,—the lawyer, whose mental toils have been incessant and unwearying, and whose labours have been crowned with all of fame that he could have dreamed of, or hoped for,—the statesman, capable, by nature, and as a consequence of hard thought and study, of wielding the interests and destinies of his country,—these men and such as these, the foremost men of the world,—among the ordinary and the sad victims of this unsparing disease; and not only these men,—but their offspring, the lineal inheritors of the organisation that prepared them physically for gaining and accomplishing so much, inherit their diseases more surely than their talents and intellectual and moral capabilities." P. 195.

Now it seems to us that, although there is a certain amount of truth in these statements, there is, at the same time, a vast deal of fallacy. The mere rank of the individual, the nobility of his blood, or the capacity and exercise of his intellectual faculties, have really very little to do with the development of gouty disease, except in an indirect and subordinate manner. We do not, as a matter of course, deny that the disposition to gout is often, very often, an hereditary penalty for the follies and vices of one's progenitors; for most truly it may be said of this malady, that "the fathers have eaten sour grapes, and their children's teeth are set on edge."

But to assert that "the higher the rank, the more pure and noble the blood, the greater the powers of mind, and the more these powers are used, ........ the more liable is a person to become the victim" of Gout, is little more than a mere flourish of words. True ; a member of the Howard or Talbot stock may be more liable to the disease than the son of an industrious tradesman, more especially if the one drinks his claret or port every day, and the other indulges in such luxuries but two or three times a year ; but then we are not to forget, at the same time, that the former, if he chooses, may very generally avoid (or nearly so) the evil, by the adoption of two very simple expedients—temperance in diet, and regular active exercise. And as to the vigorous employment of the intellectual powers being a provocative of gout, what think ye of such men as Wellington and Napoleon ? We never understood that either of them was liable to it ; and why ?—simply because both, in spite of their incessant mental occupations, were uniformly (and, thank God, one still is) most temperate and active in their mode of living. Nearly the same thing may be said of Scott, Southey, and many others, whose literary labours have been truly prodigious in point of extent. As to the constantly quoted case of Sydenham, who expressly tells us that the composition of his treatise on Gout brought on repeated attacks of the malady, be it remembered that his system at the time was, so to speak, *charged* with the morbific principle, and that little was required to cause its explosion. If studious men will neglect certain simple hygienic rules—which are obligatory upon all—and neglect, for example, to take proper exercise, or indulge unduly in the pleasures of the table, they will doubtless be more liable to gouty disorder than if they were more attentive to these rules ; but the morbid tendency is the result, not so much of the excessive intellectual exercise, as of the faulty corporeal regime. Who ever heard of a poor poet being the victim of Gout, although, day by day, he racked his brain (and even the night was not given to sleep) in giving birth to his prolific strains ; or of the weary clerk, whose pen is in his hand for 12 or 16 hours a-day, being laid up with so aristocratic a disease ?

Nay, the very exercise of the mind seems to be often antagonistic to, and in some degree a corrective of, the development of Gout in those who may be predisposed to it, whether from hereditary infection, or from intemperance of diet ; provided always due corporeal exercise be taken at the same time. What but the mental and bodily activity of such a man as Brougham, who has led anything but a Rechabite's life, has kept him in a great measure intact ? Again, it is not the post-captain, while engaged in the arduous duties of foreign service, that is apt to be laid up, but the retired commodore, who is confined to *port;* nor do we often hear of the hard-working curate, on a hundred pounds a year, having the honours of a swathed toe ; these he leaves to his sleek and stalled superior.

It must not, however, be supposed that we at all question or deny the influence of Hereditary predisposition in the production of gout. Who can be surprised at it ? Horace has said, with his usual felicity :

> Fortes creantur fortibus et bonis :
> Est in juvencis, est in equis patrum
> Virtus :—

and truly the *vice,* no less than the *virtue,* of parents is found in their offspring still more frequently among human beings, than among cows and horses. The remark holds true equally of the mind and body; and what the poet subsequently says of the culture of the one, is as applicable to the management of the other.

> Doctrina sed vim promovet insitam,
> Rectique cultus pectora (corpora) roborant:
> Utcumque defecere mores,
> Dedecorant bene nata culpæ.

As to the influence of hereditary descent in reference to gout, there is less reason for surprise, than touching perhaps any other malady to which the human frame is liable; and for this simple reason, that the offspring, besides inheriting an enfeebled constitution, too generally follow the very same habits of living that have probably engendered the disease in their progenitors. Dr. Robertson, like most other experienced physicians, calls in question the vulgar assertion that gout has a tendency to pass over one generation and re-appear in the next.

"That one or even two generations of such families do sometimes remain free from gout is certainly true; and that this immunity does not necessarily exhaust the hereditary influence, although it must greatly diminish its degree, appears to be true; but that the grandchildren of gouty people are more likely to be gouty than their children, supposing the children to continue free from the disease, is unquestionably one of the many vulgar errors." P. 10.

The Chapter on the Exciting Causes of Gout may, we think, be fairly excepted to in many particulars. Dr. R. regards atmospheric changes as the most common and influential, while irregularities of diet and excesses in the pleasures of the table are declared to be only occasional or "not uncommon" causes. We should be inclined to reverse this order of things; nor do we agree with our author in attaching more importance to the stimulant and repleting effects of wines and malt liquors, than to their tendency to generate lithic and other acids in the system. Certain it is, that it is not the spirit-drinker, but the wine-drinker that is usually the victim of Gout. The disease is of exceedingly rare occurrence in those who are much given to intemperance in the use of alcoholic liquors, even when the diet is at the same time rich and nourishing, and the blood (we may therefore reasonably suppose) is highly charged with fibrinous matter. Here then we have the very condition of the system that ought, according to Dr. Robertson's views, to give rise to the malady; but that such is not the case, we may appeal with confidence to the experience of every physician. Unless the *ingesta* are of such a nature as to be liable to create a tendency to the generation of acid matter in the stomach and bowels, they are not found, in the generality of cases, to occasion Gout. In what other way shall we explain a circumstance that is quoted by our author from Dr. Wm. Budd's treatise on the disease, in the Library of Medicine.

"There is a body of men employed on the Thames whose occupation it is to raise ballast from the bottom of the river. As this can be done only when the tide is ebbing, their hours of labour are regulated by that circumstance, and vary through every period of night and day. They work under great exposure to inclemencies of weather; their occupation requires great bodily exertion, occasioning profuse sweating and much exhaustion. In consideration of this, their allowance of liquor is very large; each man drinks from two to three gallons of porter daily, and generally a considerable quantity of spirit besides. This immoderate consumption of liquors forms the only exception, as far as relates to food, which these men offer to the general habits of the lower classes in London. Gout is remarkably frequent among them: and although not a numerous body, many of them are every year admitted to the Seamen's Hospital Ship, affected with that disease. This is a very interesting fact, and seems to show that no amount of bodily exertion is adequate to counteract the influence of such large doses of porter; the exposure of ballasters to wet and changes of temperature probably favours its operation. These men are almost all derived from the peasantry of Ireland; they can rarely, therefore, inherit a disposition to gout." P. 27.

It may, however, be fairly questioned, whether the disease among these men is genuine unsophisticated gout, or whether it does not rather partake a good deal of the nature of rheumatism.

So much for the predisposing and exciting causes of Gout. We come now to Chapter IV. which treats of "the nature, or proximate cause" of the disease. The opening paragraph is as follows:—

"Although it must be admitted that we do not know what is the proximate cause of gout, the condition with which the disease is intimately connected, and upon which its phenomena seem to be chiefly dependent, is well known. It consists in the deposition of lithic (uric) acid, and its compounds with alkalies, and principally with soda, in the fibrous tissues. It is this which serves to distinguish gout from other diseases, which is the principal feature of its morbid anatomy, and which guides its treatment, and influences its results." P. 69.

Our author afterwards, with a most teasing sort of hesitation which leads him neither wholly to adopt, nor altogether to reject any opinion, expresses himself in much less decided terms upon this etiological point; for we find him saying, that " all the phenomena of the disease do probably countenance the hypothesis, that the deposition of lithic acid always attends an attack of gout, and perhaps always precedes it. But, nevertheless, this is only hypothesis; and is only entitled to any attention, because it does not appear to contradict any of the phenomena of the disease, and because it does not militate against any one of the practical matters, which experience has shown to be useful and important, in its treatment and prevention."

Indeed!—is there not any better than this merely negative evidence to prove the intimate connection between the generation of this Acid and the development of gouty disease?

Dr. R. subsequently *appears* to shift his ground in his ætiological reasonings: for we find him, towards the conclusion of his work, expressing his belief that " gout essentially depends upon an accumulation of nitrogenised and carbonised products—which accumulation depends, in its turn, upon their inordinate supply in the form of food, or their deficient expenditure by a proportionate supply of oxygen for the elimination of the one and the combustion of the other." We regret that any sound practitioner, like our author, should have committed himself to such vain and gratuitous phantasies as these. Not only does he adduce no proof whatever that there is any such redundancy of nitrogenised and carbonised elements in the system during the existence of gout, but he must also know full well that there are various morbid states in which there is unquestionably such a redundancy, while there is not a single symptom of gouty affection present.

And here we must guard our readers against too readily admitting many of those (unquestionably most ingenious) views of disease that have been propounded by Liebig and his followers. There is a tendency in the present day to carry the love for minute chemical and microscopical observations and disquisitions to an extravagant length, and to overlook many of the more obvious and easily-recognisable phenomena of living bodies in health and in disease.

This remark suggests to our minds one or two queries, which may be fitly introduced at this part of our enquiry, with the view (we frankly confess) of eliciting a satisfactory answer. *What is the condition of the blood in Gout? Has any analysis been made of it? and, if so, in what respects has it been found to differ from healthy blood?* These are most important questions, which stand in need of solution, before we can presume to speak with any degree of precision upon the "nature or proximate cause" of the disease; and yet, as far as we are aware, there are no data whatsoever to enable us to form any warrantable opinion. In the most recent work on Hæmatology—Simon's Animal Chemistry, translated from the German by Dr. Day—there is not even a passing remark as to the state of the blood in gout. We had hoped, indeed, to have met with some observations, if not positive facts, connected with this most interesting enquiry, in an elaborate treatise upon the disease, from an author who attributes so much to the condition of the circulating fluids in its production. The only passage that at all bears upon this subject is the following:

"The character of the blood drawn can afford no guide, as to the quantity that should be taken, or as to the advisability of repeating the venesection, in any arthritic disease, and, perhaps, least of all, in gout. In all such diseases, the state of the blood is probably much influenced, and at all events, the separation of the fibrine from the colouring matter, &c., after the abstraction, is accomplished more readily, and to a greater extent, than is either natural, or than is commonly the case in other equally inflammatory affections,—giving a remarkably thick coat of fibrine to the crassamentum, leaving the lower part of the crassamentum friable or little tenacious, and separating the serum from the crassamentum more entirely, than is usual in other cases of inflammation." P. 218.

After this merely conjectural (?) description of the blood in Gout—a description which is obviously much more applicable to Rheumatism than to Gout—we are favoured with the following most edifying interrogations:

"Is this state of the blood a mere attendant, or a cause, or a consequence, of the peculiar inflammatory action? or is it mixed up intimately with the habit of body predisposing to the peculiar inflammation? Does it depend on the blood being surcharged with carbonaceous matter, from undue action of the hepatic, or deficient action of the pulmonary functions? or does it not rather depend on deficient expenditure of the fibrine itself,—the waste, and already used, fibrine of the system being either imperfectly carried off, or an undue amount of nitrogenised matters being assimilated." P. 219.

Although the condition of the Blood in gout has hitherto been so entirely overlooked, there has fortunately been a good deal of attention paid, more especially of late years, to the character and general properties of some of the chief excretions from it. Of these, none is so uniformly and so decidedly disordered as the Urine, and, perhaps we may add, the matter of Perspiration. In ninety-nine cases out of every hundred, there is an excess in the relative proportion of the Lithic acid; and in the remaining case there is probably an excess of the earthy Phosphates. What says our author upon this subject? While he admits that "the functions of the kidneys are much deranged in a large majority of gout cases, and more deranged than they are usually found to be in other disorders, which are attended with the same amount of constitutional disturbance;" he remarks, in a subsequent passage, that "the action of the kidneys is not always affected, even in acute cases of gout, nor by any means necessarily in the gouty habit of body. In some of the worst cases of Gout that I have met with, the urine has never been deranged, in quantity or character, either during or between the paroxysms. But in such cases it has been invariably true, that some other organ has suffered in the direct proportion that the kidneys have been exempted from derangement. In these cases, the hepatic functions have generally been much affected, and have been peculiarly subject to derangement; or the heart, or the skin, has been especially liable to be disordered."

We must confess that we have our doubts as to the accuracy of Dr. Robertson's observations in the cases to which he alludes; and the more especially as it is well known that the urinary secretion is very rarely natural, whenever the hepatic functions are much disordered. Dr. Prout has pointed out the frequent connection of several biliary disorders with a tendency to lithic acid deposits in the urine.

With respect to the excretion from the skin, our author states that it "is often odorous and strongly saline, and is perhaps never in a healthy state during the paroxysm" of gout: he admits that it is occasionally acid. May we not, however, fairly presume that these statements are not so much the results of actual experiment, as the suggestions of what is most probably the case?

We wish now to draw the reader's attention to two not uncommon, although often misunderstood, forms of Gouty disease; more especially as their history, therapeutic as well as ætiological, seems to us to illustrate and give sanction to some of the sentiments expressed in the preceding pages; and first of

*Gouty Catarrh.*

This is a peculiar description of bronchial irritation which is apt to occur in certain gouty constitutions (although perhaps there has never been any *open* development or invasion of the disease), and is very well described in the following passage.

It is "a condition characterised more by symptoms of morbid sensibility, than by those of undue excitement, but which sometimes takes on, and perhaps in all cases is disposed to take on, a more decidedly bronchitic character. This affection is marked by a peculiar dry and wheezing cough, usually occurring in paroxysms, which end generally in more or less of expectoration. This cough is more common in gouty people as life advances, for the obvious reason that old people are more subject than the young to bronchial affections. This affection is usually characterised by being little amenable to ordinary treatment, and more influenced by the condition of the gouty joints, than by the usual means by which bronchial affections are relieved or removed.

"Although thus closely connected with gout, and dependent upon it, this bronchial affection is, nevertheless, often distinctly traceable to cold as its exciting cause; and cold and wet may exacerbate this, as any other form of bronchial irritation. When it takes on an inflammatory character, this affection must, of course, be subjected to the same treatment, as the bronchitic symptoms would otherwise indicate; but if the case does not assume an inflammatory character, the usual treatment of bronchial affections is in general of little or no use. The bronchial affection increases or diminishes with the fall or the increase of the localised gout, and is seldom got rid of for any great length of time, without a fit of gout in the extremities: unless, perhaps, during the summer months, when the increased action of the skin, and the less risk that the degree of its action may be suddenly diminished, lessens materially the amount of work to be done by the bronchial mucous membrane, and the risk of variation in the amount of its duties, thus removing a principal exciting cause of this morbid condition. It should be borne in mind, that, although not of common occurrence, this affection may herald, or even attend, the supervention of phthisis on the gouty constitution." P. 176.

This very troublesome, and generally most unmanageable, form of Catarrh is sometimes met with in persons who have never suffered from any obvious attack of gout, and do not inherit any tendency to the disease. If asked whether they have anything gouty about them, they will confidently reply in the negative; and, it may be, justly so. But then it will be found almost invariably that they have lived upon the most approved plan of generating the gout; viz. indulging their appetites, and drinking their three or four glasses of port wine daily. This is very generally the secret of the malady; and hence it is that, in nineteen out of every twenty such cases, very decided relief, if not a cure, may be effected by a mere change in the diet, after all the cough medicines in the world have been tried in vain, and the patient perhaps has been put to the expense and annoyance of travelling to a warmer climate to seek for a cure. Alkaline medicines are always useful in such cases; the proper time for taking them is usually from one to three hours after eating. Before quitting the subject of this cough, it may be worth while to remark that its severity is generally not a little influenced by the *temper* of the individual: *non rectius podagræ, quam iracundiæ, paroxysmus dici potest.* Hence it is apt to vary much on different days, quite independently of the weather; it is, moreover, always less severe when the mind is amused.

### *Gouty Neuralgia.*

Upon this subject, Dr. Robertson remarks:—

"The sheath of the nerves is not an unusual seat of irregular gout, forming what is called gouty neuralgia. This is generally a very severe and intractable complaint. It is probably the worst and most obstinate form of neuralgia, that does not depend upon organic changes, and is not therefore incurable. The history of the case—the individual having suffered from gout, or being predisposed to it, or having led such a life as to lead to the inference that the predisposition may have been induced—especially if strengthened by the presence of some symptoms of irregular gout, would serve fully to diagnose a case of this kind. The best and most speedy cure of this affection is to be found in a fit of regular gout. It may exist, in despite of all treatment, for years. It may have little effect on the general health; or it may derange it essentially, and superinduce serious or fatal disease,—most usually, as it has seemed, of the heart. Like all the other forms of irregular gout, it is, in most cases, traceable to the suppression, or retrocession, or imperfect development of the disease, and is very generally mixed up with general debility. The sciatic nerve is by far the most common seat of this form of gout; and sciatic gout is the name by which it is commonly distinguished; but occasionally, although very much more rarely, the nerves of the face, or of the upper extremity, are affected by it. That it is essentially gouty in its nature, is, perhaps, sufficiently proved by its history, its *irregularly* paroxysmal character, its origin in the gouty system, and more or less directly from imperfectly developed gout, and by the immediate relief obtained by a developed fit of gout. In general, this form of neuralgia is less influenced by change of weather than ordinary neuralgia; but there are too many exceptions to this, to make it of decided value as a means of diagnosis. If not the most immediately alarming form of gout, it is very tedious, uncertain, and difficult of management, and one that often exhausts the patience of the sufferer, if not of his medical attendant. A thorough change of habits, mode of life, place, kind of air and scene, is almost always indispensable to any hopeful treatment of this affection; and the occurrence of gout in some other tissue is what is to be most wished for." P. 190.

That many cases of Neuralgia are connected with a gouty diathesis, or, in other words, with the existence in the system of that peccant material which is always present in gout, will be admitted, we should think, by most practical men. So far, Dr. Robertson is, in our opinion, perfectly right; but we cannot agree with him that " the best and most speedy cure of this affection is to be found in a fit of irregular gout," or " that the occurrence of gout in some other tissue is what is to be most wished for." Why so?—if the cause of the neuralgic suffering be known, and if we possess remedies, the judicious employment of which will almost infallibly neutralise and perhaps altogether subdue the operation of this morbific cause, why should we desire that our patient may have an attack of regular gout? Such a desire is only to be entertained, one might fancy, in those cases where the physician has overlooked the real origin of his patient's malady, and where, therefore, the obvious development of the gouty disease, in the foot for example, will have the effect of opening his eyes to the true nature of the case.

The following case of *gouty neuralgia of the intestines,* which occurred in our practice several years ago, may be aptly quoted here. A gentleman, 60 years of age or thereabouts, and who, twenty or thirty years before, had suffered for a length of time with that form of Bronchial Irritation to which we alluded in a preceding page, had, at intervals of various duration, an attack of severe pain in the left iliac region; this part became exceedingly tender on pressure, so that it was generally deemed advisable to leech and foment the part, as well as to administer antiphlogistic medicines to keep down the febrile irritation that was present. The attack usually lasted for three or four days; but a certain degree of tenderness remained in the groin for several days longer. Several medical men had visited him, and more than one suspected that there was incipient organic mischief forming in the descending portion of the colon. If so, how came it to pass that the attacks were (irregularly) periodic, and that, in the intervals between them, the patient was quite exempt from all uneasiness? Upon the urine being tested in one of these intervals, it was found to be decidedly more acid than in health; and, coupling this fact with the circumstance that the patient had at a former period of life suffered from a gouty complaint, we deemed it advisable to put him upon a course of alkaline medicines, and prohibit the use of all vinous and malt liquors. The result was most gratifying. For the next twelvemonth, he remained almost entirely exempt from a return of the abdominal attacks.

We come now to a point in the history of gout that deserves much attention, and which, although it has been often discussed, is far from being well understood, or definitively settled—we allude to *the diagnosis between Gout and Rheumatism,* more especially between the chronic forms of the two diseases.

Our author at first admits that the diagnosis is often " difficult and sometimes undecidable ;" for he says :

" In both, gout or rheumatism may have been unknown, or unheard of, in the preceding generations of the family, or both may be believed to have been present ; in both, the age may be the same ; in both, the habit may be equally plethoric, or equally cachectic ; in both, neither rheumatism nor gout may have been felt in the passed years of life ; in both, the urine may indicate undue acidity, or not ; in both, the stomach may be out of order, or the digestion little affected ; in both, there may only occasionally be felt any pain or uneasiness of the affected parts, which may have become gradually swelled, stiffened, charged with hard deposition, and contracted ; and such cases may, indeed, often be diagnosed with much difficulty." P. 206.

Yet, after this alleged most striking family likeness, the two maladies are declared to be essentially different and distinct from each other. Is there not some incongruity in all this ? and is not the incongruity rendered still more remarkable by the circumstance that, among the diagnostic marks enumerated as existing between the two diseases, we now find our author expressly making mention of gout as a " complaint untraceable to cold and very little, if at all, aggravated by change of weather ;" whereas, in the very first sentence of his third chapter we read, " of the exciting causes of gout, those which act the most commonly and the most influentially, are changes, and especially sudden and great changes, in the temperature, &c., of the air."

That there is a difference between Rheumatic and Gouty disease, no one can reasonably deny ; but certain it is that Dr. R. has not assisted us in discovering in what the real and essential difference lies. Nay, has he not added somewhat to the difficulty by the very pathological views as to the nature and proximate cause of gout, which he so continually brings before the eye of the reader ?—We allude, as a matter of course, to the doctrine that it is a disease " based upon plethora, and consists essentially in a redundance of nitrogenised products in the system." Does not this description apply with much greater accuracy to Rheumatism than to Gout ? It is a fact beyond all question that the former malady is especially apt to occur in plethoric constitutions, and that, in it, the blood is very generally charged with an excess of its fibrinous constituent. Holding the views which our author does, we have been not a little surprised to find that he repudiates the idea of gouty and rheumatic disease being almost ever combined in the same case. " As to the disease called rheumatic gout, the name is often used as an excuse for uncertain diagnosis, and applied sometimes to gout, and sometimes to rheumatism ; the mixing-up of the two diseases in the same case being, in truth, of very rare occurrence ;" and, in the Preface to his work, he goes so far as to say : " I trust that the time will come when the so-called disease, *rheumatic gout,* will be as seldom heard of, as I believe it is of rare occurrence." We are utterly opposed to the admission of Dr. Robertson's opinion upon this very important point—important, because, according to the views that are held, so will be the medical practice that is pursued in a multitude of cases. That gouty maladies are often engrafted upon, or associated with, a rheumatic constitution cannot surely be disputed by any one; we should say that they are very generally so in patients who are young, plethoric, and indulge much in the use of fermented liquors; simple uncomplicated gout being comparatively rare in such persons. But it is quite unnecessary to enlarge upon this subject, unless we have previously determined what Gout and Rheumatism really and truly are. Dr. Robertson does not say a single word as respects the proximate cause of the latter disease; and as to that of the former, we have already seen how very questionable some of his opinions are. We cannot, as a matter of course, enter at large upon this question. Suffice it to say that, after the most mature consideration of the subject,—based not only upon what has been written by the most experienced authors, but also upon the results of minute observation in practice—we have long since come to the conclusion that genuine uncomplicated rheumatism, whether it be of the acute or chronic form, is always connected with an excess or redundancy of the fibrinous constituent of the blood. Many circumstances seem to favour this idea. The disease very generally occurs in persons of a plethoric habit of body, and who are liable to inflammatory disorders, either from a congenital predisposition, or from an excessive consumption of strong and nutritious food. The state of the blood too, when drawn, very generally shews that there is a redundancy of fibrine; and when any serous surface becomes the seat of the rheumatic inflammation, it is well known that there is almost invariably an effusion of coagulable lymph. Moreover, what is the medicine which of all others appears to have the most prompt and decided effect, not only in causing the absorption of the lymph that has been already effused, but also in defibrinating (so to speak) the condition of the blood? Is it not Mercury? And is not this the very remedy on which we can best depend for the relief of many of the most serious and distressing symptoms of Rheumatism? If the system once becomes gently mercurialised, not only do the present sufferings of the patient almost immediately subside, but the existence of any internal mischief is generally counteracted and subdued.

In saying this, however, we do not wish it to be understood that the treatment of Rheumatism should be left entirely to the exhibition of mercury. Moderate venesection may be, and very often is, unquestionably useful; it controuls excessive febrile action, and at the same time powerfully promotes the curative operation of other remedies. Then, again, the secretion of the kidneys is almost always very sensibly deranged; usually it is sparing in quantity, and also highly charged with its peculiar principles, which, if not duly eliminated from the system, invariably aggravate all pyrexial irritation.

Whatever may be said of the theory we advocate, we can vouch for the very general success which will attend the practice that is based upon and derived from it: viz. the use of a mild course of mercurial alteratives, and of saline, and especially alkaline, diuretics; the occasional moderate detraction of blood, either generally or locally; a light and lowly animalised diet; and a most rigid abstinence from all fermented liquors, more especially from those which contain much glutinous and nutritious principles, such as porter and ale. We have almost daily occasion to witness the good effects of entirely prohibiting all such stimulating drinks in chronic rheumatic ailments, and we are not unfrequently surprised to find that many medical men seem to neglect so much the regulation of their patient's diet, while cramming them with physic;—the very virtues of which are often directly counteracted by the pernicious *ingesta*, that are allowed to be taken at the same time. We need scarcely say that warm clothing is another essential adjuvant in the treatment of all cases of rheumatism. Its use is the more necessary, when we administer mercury and diuretics; as the action of these remedies invariably renders the cutaneous surface more sensitive to atmospheric impressions than it was before.

If the view which we have now taken of Rheumatism be correct, it is sufficiently obvious that the morbid condition of the blood, upon which it is believed to depend, may be associated with a cotemporaneous dyscrasis of the circulating fluid of a different nature. Along with the excess of fibrine, there may be, for example, the presence of an abnormal acid matter, such as we have every reason to believe exists in genuine and uncomplicated gout. If such be the case, may we not reasonably suppose that the phenomena, to which this compound morbid state may give rise, will have a compound character, partly rheumatic and partly gouty? Such is the view that we take of what is called "rheumatic gout"—a malady, we feel assured, that is of very common occurrence, in spite of the repeated contradiction of our author.

We have not left much space to ourselves for the examination of the therapeutic portion of Dr. Robertson's work; but we must allude to some of the more salient points.

He is unfavourable to the application of leeches to a part affected with gouty inflammation: "it is hardly ever of the least service, and is, very generally, decidedly injurious to gout. If applied near the part affected, the local inflammation is usually increased, or at all events unrelieved, by them; and if applied at a distance from the local gouty inflammation, there is considerable risk, that the gout will either migrate to that neighbourhood, or make its appearance there in addition to its existing site. There is, probably, one description of cases, in which the application of leeches might be of much service, and in which it would be advisable to give them a trial. This is when gout has taken up its seat in some very undesirable position, as in the loins or hip, and when the amount of febrile and inflammatory action seems otherwise to indicate the propriety of loss of blood, and the other circumstances of the case do not contra-indicate it, and when it seems not unlikely that the application of leeches to the more common and desirable seats of gout, as to the great toe, might be attended with good effects, and lead to the migration of the disease, and determine it to the part where the leeches had been applied."

In our opinion, there is a great deal of unnecessary apprehension in the minds of many medical men about the effects of local bloodletting in gout. As a matter of course, there is no use of having recourse to it at all, unless the inflammation in the part affected be tolerably active and severe; but, whenever this is the case, the application of a few leeches in the neighbourhood of the inflamed joint will very generally afford decided relief. The number of leeches need never be great; a gradual oozing from a few bites, kept up for several hours, is always much more beneficial than the more rapid depletion from a multitude of them. The best plan to promote the gentle bleeding is by covering the affected part with flannel wrung out of hot poppy-head fomentation, placing over this a piece of oil-skin. The part should never be exposed to the air. On the following day or so, the use of a weak spirit lotion, *applied tepid*, will generally be both pleasant and beneficial. One part of spirit to three of camphor mixture is the application so much recommended by Sir C. Scudamore. The addition of a little vinegar will often be found very grateful.

*Colchicum* is declared by Dr. Robertson to be "decidedly an evacuant, and as decidedly a narcotic." Although most writers have alleged that this plant possesses distinct *narcotic* properties, we are by no means satisfied of the correctness of the assertion. That its use will often quiet pain, more especially that of Gout and Rheumatism, and that it is, therefore, really and truly a *sedative*, no one can deny; but that it ever produces any decided narcotism, or stupor, we very much doubt. Its operation is in many respects very similar to tbat of antimony; both acting upon the stomach and bowels, the skin, the nervous and muscuular systems, and more particularly upon the heart and blood-vessels, in very nearly the same way. That its "virtues as a gout-medicine are unquestionably owing to its specific effect upon the fibrous tissues," is a very vague and undemonstrable assertion. As to the conjecture of our author that it probably contains "three differently acting principles, one being evacuant, a second narcotic and a third sedative," we deem it most improbable. The *primary* effects of the medicine seem to be exerted upon the mucous surface of the stomach and bowels, stimulating their secretions, and powerfully affecting their nerves, (in a manner, there is reason to believe, similar to that of Veratria upon the skin); its *secondary*, are directed more immediately upon the kidneys, exciting them to a more active elimination of lithic acid, and probably too of other nitrogenised elements, from the system. The action of the remedy is therefore twofold; first on those organs engaged in what Dr. Prout has called the process of *primary assimilation*, and afterwards on some engaged in the process of *secondary assimilation*. According to this view, Colchicum ought to be very useful in many other maladies besides Gout and Rheumatism; and so it unquestionably is. We have found it often produce excellent effects in various cerebral and hepatic disorders, more especially when these occur in full and plethoric habits. In the early stage of acute Hydrocephalus, it may be given with great advantage; and, in short, in almost every disease, in which we wish to make a strong impression on the intestinal canal and on the kidneys. As a general remark, it may be well to state that, whenever we have reason to suspect that the bowels are loaded or the intestinal secretions much depraved, the use of colchicum should always be preceded by some active purgatives in combination with a mercurial. This is just what might have been anticipated, considering the *modus operandi* of the drug. By its peculiar influence on the intestinal surface, it acts as a powerful derivative of the distal irritation that is present in the hands or feet; and, if this revulsion take place before the bowels have been cleared of their offensive contents, the morbid action may fasten itself upon some vital organ, and give rise to the most alarming symptoms. Whoever has tried upon his skin the effects of Veratria (and the *veratrum* belongs to the same natural family as the *colchicum*), will remember the peculiar effects which it has upon the cutaneous nerves; and the continued tingling irritation, which it produces, has, it is well known, often relieved the most painful neuralgia—although there has been no narcotic nor directly sedative operation experienced, as when the active principle of Aconite, and such like plants, has been used. Now, is it not possible that Colchicum may act in a somewhat similar manner upon the nerves of the alimentary canal? and, if it does, can we be surprised at the pernicious effects which it may occasion, while the *materies* morbi is yet uneliminated from the system.

The too-common practice of calling Colchicum a specific for gout has given rise to the most unscientific, and, unfortunately too, the most pernicious errors. Its use may relieve pain, and it may powerfully assist in promoting the expulsion from the body of the peccant matter that is invariably present to a greater or less extent; but it will never by itself serve to prevent the new formation of this matter, nor can it even cure some of the worst sufferings to which gouty patients are liable. We cannot therefore go so far as Dr. Robertson, and admit with him that "so much power has this wonderful drug on gouty action, that it enables us, in a very great degree, to prevent, relieve, or modify its manifestation."

Fortunately, the use of this potent remedy is not necessary in a vast number of cases of Chronic Gout. The exhibition, continued for some time, of mild alkalis, and the regulation of the diet, so as to prevent as much as possible the formation of acid during the process of digestion, with due attention to the state of the alvine excretions, will do more for the effectual and permanent relief of our patient, than the use of Colchicum, or of any boasted specific in the world.

With respect to the employment of Colchicum in Rheumatism, the following passage will sufficiently explain our author's sentiments.

"The degree to which colchicum may and must be given in rheumatism, especially articular rheumatism, and more especially when the fibrous tissues of the heart are involved in the inflammatory action; the promptness with which it should follow the preliminary doses of calomel and purgatives; the steadiness and freedom with which it should be exhibited; the length of time during which it should be continued, in lessening doses, until the heart affection and the state of the joints, evidently render it no longer needful; and that colchicum should be made the sheet-anchor in these cases, and not calomel and the lancet,—these being auxiliary, and only auxiliary, to the specific means of relieving fibrous inflammation; these are truths that will, every year, be known and felt more and more." P. 256.

Few medical men, we should think, will agree with Dr. R. in his estimate of the comparative value of Colchicum and Mercury, along with bloodletting, in rheumatic Carditis.

We must not omit to give our author's opinion of Iodine in the treatment of Gout.

"The external or internal use of iodine in the different forms of gout, during the absence of acute or active inflammation, is a question of rising interest. That it does not debilitate the system, or at least, when carefully given, does not debilitate it to the degree that was at one time feared or conceived, is now generally admitted. On the contrary, many persons evidently become strong, and rapidly gain flesh and power under its use. That it has great alterative powers, is now admitted on all hands; that it acts almost specifically on the glandular system, and the emulging organs generally, might lead to an inference, as to its possible usefulness in gout; and there can be little question, that, in many chronic, and no small proportion of irregular, gout cases,—and in many cases of confirmed gout, when carefully used between the paroxysms, its effect is most useful; the more so, of course, other things being equal, the more cachectic the general habit, or the more that a scrofulous condition seems to be mixed up with gout. In these, and indeed in all cases, too much cannot be said about exhibiting iodine in mild enough form, and small enough doses; preferring the iodide of potassium until it is found, on trial, to be insufficient." 310.

The following passage struck us as deserving of notice, rather from the singularity than the soundness of the opinion therein expressed.

"It is believed (by whom?—*Rev.*) that a new era of gout-treatment will mainly consist, in the greater trust in mercurials, and diaphoretics, for the relief of the inflammatory condition,—the much diminished use of colchicum,—and the greater and greater use of alterative means, and, perhaps, chiefly of iodine, to correct the habit of system on which gout mainly depends, or by which it is always much aggravated in its degree, and lowered in its character, and seriously exacerbated in its consequences." P. 310.

From the remark in the closing part of this sentence, it would seem (as indeed we had reason to suspect from a previous part of his work) that Dr. R. regards the gouty cachexia to be of a strumous or semi-strumous character—an opinion that is not likely to be generally entertained.

A much more useful remedy than *iodine* as an alterative or corrigent of the gouty diathesis, in aged and infirm persons, is *steel*. The milder preparations of this metal—and none is better than the sesqui-oxide, as Dr. R. very judiciously remarks—will, in very numerous cases of Irregular Gout, be found of the greatest service; after the alvine secretions have been attended to, and the urine has been brought back to a healthy condition by the use of alkaline medicines. A course of gentle chalybeates will then serve, better than any other tonics, to invigorate the digestive organs, to counteract the tendency to mal-assimilation of the food, and to give tone to the general capillary system. We have repeatedly witnessed admirable effects from such a course of treatment.

*Arsenic* also has been administered in such cases with decided benefit; especially, as our author observes, when the disease exhibits more than usual of the paroxysmal character. Change of air, and the occasional use of some of the native mineral waters, will generally much promote the efficacy of the remedies employed.

The great diminution, in point of frequency, of Gout during the last 30 years in this country, is obviously attributable to the much greater temperance in the use of vinous liquors that has prevailed since the close of the last war. Men now-a-days eat quite as much as ever they did, and are, we verily believe, quite as plethoric as they used to be; but they certainly do not ingurgitate so much Port wine—the true "fons et origo" of a vast deal of gouty disease. Persons, who have a tendency to the malady, and who yet have not the control over their appetites to resist the use of vinous and malt liquors, should be instructed to take a scruple of carbonate of soda or a little calcined magnesia about two hours after their dinner: this will serve to neutralise the acidity that is developed during the process of digestion. Sir H. Halford was much in the habit of prescribing a rhubarb and soda pill, forenoon and evening, to his gouty patients: the practice is a very good one. The stomachic remedy well known as Gregory's powder—consisting of rhubarb, magnesia, and ginger—is also very serviceable, if taken an hour or two after meals. It should ever be remembered, that the foundation of all rational treatment of the gouty diathesis must be the correction of the disordered assimilative functions, and the neutralization of the peccant acid matter already introduced into the system;—the intestinal and renal excretions having been first brought into a healthy state. The fountain-head of the evil is in the chylopoietic viscera; if the spring be troubled, how shall the streams be pure? To ascertain this cardinal point, the condition of the alvine and urinary evacuations must be known; such knowledge is as necessary for the judicious treatment of Gout, as that furnished by the auscultatory exploration of the chest is for the treatment of Dyspnœa or acute Rheumatism. Unless the physician makes himself, every now and then, acquainted with the chemical properties of the urine, his practice *must* be empirical, and *may* be often most injurious.

But, besides the secretional disorders alluded to, there may be superadded other morbid conditions of the system in cases of gouty disease; and to these also, as a matter of course, proper attention must be paid. There may, for example, be a plethora of the vessels, and the blood may be, at the same time, unduly rich and fibrinous; or there may be a highly excitable condition of the nervous system, and a consequent proclivity to the development of various spasmodic and neuralgic symptoms. Again, the hereditary constitution of the patient should be studied; the maladies from which he has previously suffered; the mode of life he has followed; the season of the year; the medical constitution of the atmosphere, and so-forth. When we think of all these things, is it not obvious that the same line of Treatment cannot possibly be appropriate in every one of those various, and often too very different, maladies that have been so unnaturally forced together and congregated under one general appellation? At all events, let not the practice that is followed be regulated by a mere name, and directed against a shifting phantom; but let all the existing phenomena in each individual case be diligently and minutely investigated for themselves. Then will the medical treatment be alike more rational and successful; we shall no more hear of *specifics* for Gout.

From the preceding pages, the reader will doubtless be enabled to judge for himself of the general character of Dr. Robertson's work. We have freely expressed our own opinion, and have canvassed its merits without partiality or prejudice. The therapeutic directions for the treatment and prevention of Gout are, on the whole, excellent, and may therefore be read with advantage by every one.

